# Podoplanin expressing macrophages and their involvement in tertiary lymphoid structures in mouse models of Sjögren’s disease

**DOI:** 10.3389/fimmu.2024.1455238

**Published:** 2024-09-17

**Authors:** Aud-Malin Karlsson Hovd, Saba Nayar, Charlotte G. Smith, Premasany Kanapathippillai, Valentina Iannizzotto, Francesca Barone, Kristin Andreassen Fenton, Hege Lynum Pedersen

**Affiliations:** ^1^ Department of Medical Biology, Faculty of Health Sciences, UiT The Arctic University of Norway, Tromsø, Norway; ^2^ Institute of Inflammation and Ageing, University of Birmingham, Birmingham, United Kingdom

**Keywords:** Sjögren, macrophages, CX3CR1, podoplanin, mannose receptor, CD206, autoimmunity, salivary gland

## Abstract

Tertiary lymphoid structures (TLSs) are formed in tissues targeted by chronic inflammation processes, such as infection and autoimmunity. In Sjögren’s disease, the organization of immune cells into TLS is an important part of disease progression. Here, we investigated the dynamics of tissue resident macrophages in the induction and expansion of salivary gland TLS. We induced Sjögren’s disease by cannulation of the submandibular glands of C57BL/6J mice with LucAdV5. In salivary gland tissues from these mice, we analyzed the different macrophage populations prior to cannulation on day 0 and on day 2, 5, 8, 16 and 23 post-infection using multicolored flow cytometry, mRNA gene analysis, and histological evaluation of tissue specific macrophages. The histological localization of macrophages in the LucAdV5 induced inflamed salivary glands was compared to salivary glands of NZBW/F1 lupus prone mice, a spontaneous mouse model of Sjögren’s disease. The evaluation of the dynamics and changes in macrophage phenotype revealed that the podoplanin (PDPN) expressing CX3CR1^+^ macrophage population was increased in the salivary gland tissue during LucAdV5 induced inflammation. This PDPN^+^ CX3CR1^+^ macrophage population was, together with PDPN^+^CD206^+^ macrophages, observed to be localized in the parenchyma during the acute inflammation phase as well as surrounding the TLS structure in the later stages of inflammation. This suggests a dual role of tissue resident macrophages, contributing to both proinflammatory and anti-inflammatory processes, as well as their possible interactions with other immune cells within the inflamed tissue. These macrophages may be involved with lymphoid neogenesis, which is associated with disease severity and progression. In conclusion, our study substantiates the involvement of proinflammatory and regulatory macrophages in autoimmune pathology and underlines the possible multifaceted functions of macrophages in lymphoid cell organization.

## Introduction

Primary Sjögren’s disease is one of the most common autoimmune rheumatic diseases affecting the population, with a prevalence of 0,5% to 1%. Typical patients are women in their postmenopausal stage (40-60 years old) (1, 2). The disease is characterized by lymphocyte infiltration into the exocrine glands, such as the salivary and lacrimal glands, as observed by the clinical symptoms of oral and ocular dryness (1, 3, 4). Primary Sjögren’s disease occurs in the absence of other autoimmune diseases while secondary Sjögren’s disease is associated with other autoimmune diseases, such as systemic lupus erythematosus (SLE) and rheumatoid arthritis (RA) (2). The pathology of Sjögren’s disease is complex and can be combined with several factors, in which both endogenous and exogenous factors, including dysregulation of salivary gland epithelial cells, contribute to the initial phase. In addition, activation of the immune system and B-cell hyperactivity will lead to chronic inflammation (2). The combination of several factors has a culminating effect on the destruction of exocrine tissues, such as salivary gland architecture and the development of severe dryness of the eye and mouth (2). In addition, the organization of immune cells into structures known as tertiary lymphoid structures (TLSs) within inflamed exocrine tissue is an important part of the pathology of Sjögren’s disease (5–8).

TLSs are accumulations of lymphoid, myeloid and stromal cells in nonlymphoid tissues. These structures are often observed in response to environmental stimuli and/or the transition from acute to chronic inflammation (9, 10). TLSs have been observed in most tissues in autoimmune diseases (11), infections (12) and cancer (13). They share many properties with secondary lymphoid organs (SLOs), such as the spleen and lymph nodes, in terms of their cellular composition, function and regulation (9, 10). TLSs can be the site of both immune response induction and regulation owing to the presence of structures and cells involved in both antigen presentation and tolerance induction. Regarding lymph nodes, TLSs consist of separate T- and B-cell zones, stromal cells, high endothelial venules (HEVs) and lymphatic vessels (9, 10, 14). The organization of macrophages has not yet been described in TLS.

Recent studies have shown that CX3CR1^+^ myeloid cells are involved in both Th17 and regulatory T-cell responses to infection in the gut (15). These findings might be linked to the observation that CX3CR1^+^ macrophages are involved in TLS formation in the gut (16) and pancreas (17). The mannose receptor (CD206) is strongly expressed in subcapsular sinus and medullary sinus macrophages in the spleen and lymph nodes (18). This receptor is also important in tissues and many tissue-specific macrophages express it during homeostasis (19). The polarization of monocyte-derived macrophages towards the M2-phenotype is often linked to macrophages expressing CD206. As for tissue-resident macrophages, these M2 macrophages also have a high phagocytic capacity and are often linked to anti-inflammatory processes in an immune response with the production of interleukin (IL)-10 and TGF-β (19). The formation and expansion of lymphoid tissue have been shown to be dependent on podoplanin (PDPN) expression by stromal cells (14).

In our study, we observed the dynamics of macrophage populations during the induction and expansion of salivary gland TLSs. The focus was to observe the organization of macrophages expressing CX3CR1, mannose receptor CD206, and PDPN. The expression of CX3CR1 is often cell-type-specific in tissue. Macrophage populations were characterized and observed in two different mouse models of Sjögren’s disease. A LucAdV5 virus-induced mouse model of primary Sjögren’s disease was used to follow myeloid cell populations from the acute inflammation to the resolution stage of inflammation. To compare the organization of macrophages in tissue with a non-virus induced model of Sjögren’s disease, the NZBW-F1 mouse model which spontaneously develops a Sjögren’s disease-like disease was used (20, 21).

## Materials and methods

### Mice and induction of Sjögren’s disease like TLSs with cannulation of the LucAdV5 virus

C57BL/6J mice were purchased from Charles River (#632) and maintained under specific pathogen-free conditions in the BMSU at the University of Birmingham according to Home Office and local ethics committee regulations (P4B291FAA). Hybrid (NZBxNZW)F1 mice were purchased from Jax (NZBWF1/J, #100008) and housed at the University of Toronto animal facilities under specific pathogen–free conditions, in a closed caging system with a 12-hour light/12-hour dark cycle. They were provided with a standard irradiated chow diet (Teklad; Envigo, 2918) and acidified water (reverse osmosis and ultraviolet sterilized). All animal experiments with the (NZBxNZW)F1 mice were conducted with ethical approval from the University of Toronto, Faculty of Medicine animal care committee. All mice used were 8-12 weeks old at the start of the experiment.

Primary Sjögren’s disease was induced in C57BL/6J WT mice (n=15) by infecting the submandibular gland with replication-deficient adenovirus carrying the *luciferase* gene via cannulation (22). For cannulation of the salivary glands, a glass pipette was be inserted into the oral orifice of the submandibular gland under general anesthetic (medetomidine and ketamine) intra-peritoneally. The mice were administered buprenorphine subcutaneously at least 30 minutes before the start of cannulation. Both glands were injected with 10^9^ pfu replication-deficient adenovirus carrying the *luciferase* gene via cannulation. After delivery of the virus, the cannula was removed, and the mice were subcutaneously administered atipamezole and fluids to reverse anesthesia. In addition, the mice were administered fluids at the end of the day. The mice were euthanized on day 2, 5, 8, 15 and 23 post-cannulation, with three mice at each time point. Uninfected C57BL/6J WT mice were used as controls (n=3).

### Preparation of tissue for analysis with flow cytometry

#### Digestion of salivary glands

Submandibular salivary glands from virus-cannulated and control mice were harvested. Approximately 15–20 mg of tissue was digested from each salivary gland. The tissue was chopped into small pieces and incubated with 2mL mix RPMI 1640 medium (Sigma) containing 2% fetal calf serum (FCS), collagenase Dispase (0.8mg/mL, Sigma), Collagenase P (0.2mg/mL, Sigma) and DNase-I (0.1mg/mL, Sigma) at 37°C for 20 minutes. The suspension was then pipetted up and down for 30seconds to break the cell aggregates. After the fragments had settled, 1.5mL of the digested tissue solutions were passed through a 70µm cell strainer into a 50mL falcon tube with 5mL cold 2mM EDTA-PBS buffer placed on ice. The remaining tissue fragments were further digested for 10 minutes at 37°C with 2mL medium containing collagenase Dispase (0.8mg/mL, Sigma), Collagenase P (0.2mg/mL, Sigma) and DNase I (0.1mg/mL, Sigma). The suspension was then pipetted up and down to break up the remaining aggregates before the suspension was passed through a 70µm cell strainer into a 50mL tube containing the rest of the digested tissue suspension. The filter was then rinsed with 10mL of cold 2mM EDTA-PBS buffer and centrifuged at 600g for 4 minutes at 4°C. Cell pellets were washed with 5mL of 2mM EDTA-PBS buffer and resuspended in 5mL EDTA-PBS buffer before staining with antibodies for flow cytometry.

#### Antibody staining for flow cytometry

Digested cell suspensions were centrifuged, and cell pellets were resuspended in 400µL of viability dye (1:600 Zombie Aqua™ Fixable Viability Kit, BioLegend). The cells were incubated in the dark with the viability dye for 15 minutes at room temperature and then for 10 minutes at 4°C, before 1mL PBS-2mM EDTA buffer was added, and the cells were centrifuged. Cell pellets were washed with 1mL staining buffer (containing phosphate-buffered saline (PBS), pH 7.2, 0.5% bovine serum albumin (BSA) and 2 mM EDTA) prior to antibody staining. Cell suspensions were divided into two separate flow cytometry panels, centrifuged, and the cell pellets were resuspended in 100µL of antibody cocktail diluted in staining buffer with FC-block (14-0161-85, Invitrogen, Thermo Fisher Scientific) (see **Supplementary Table 1**) and incubated for 30 minutes at 4°C. Cells were then washed with staining buffer, fixated with 400µL eBioscience fixation solution (00-5521-00, Invitrogen by Thermo Fisher Scientific) for 30 minutes at 4°C and washed with 1mL 1xPermeabilization/fixation buffer (00-8333-56, Invitrogen by Thermo Fisher Scientific). The samples were resuspended in 350µL staining buffer and kept at 4°C until sample acquisition. The samples were acquired using a Fortessa X20 (BD Bioscience). To optimally compensate for the fluorescence spillover from fluorochrome-conjugated antibodies, the VersaComp Antibody capture bead kit (B22804, Beckman Coulter) was used.

#### Analysis of data obtained by flow cytometry

Flow cytometric data were analyzed using the FlowJo software (V10.8.1, FlowJo LLC). A combination of conventional gating and t-distributed stochastic neighbor embedding (tSNE) plot analysis for clusters was applied. The data were extracted as frequencies of myeloid cell populations, frequencies of the parent gate or cell counts. Data obtained from both salivary glands were averaged to determine the condition of each mouse. Two-way ANOVA with Dunnett’s multiple comparison tests were applied to test for statistical significance in GraphPad Prism 7.05.

### Histological investigation by immunofluorescence

#### Virus-cannulated and control C57BL/6J mouse

Salivary glands from virus-cannulated and control C57BL/6J mice were embedded in OCT (4583, Sakura Finetek, USA) and frozen at -80°C. Frozen 5µm cryo-sections were cut using Cryostar NX70 cryostat (Thermo Fisher Scientific), dried overnight at room temperature and the next day they were wrapped in aluminum foil and stored in -80°C until use. For further analysis, the sections were thawed at room temperature for 30 minutes and then fixated in 3,7%paraformaldehyde-PBS for 5 minutes at room temperature, before being washed in PBS. For immunofluorescence labelling of the OCT-embedded salivary gland tissues of LucAdV5 infected C57BL/6J mice, all antibodies were diluted in PBS containing 10%goat serum and 1%BSA. To block nonspecific protein binding of the antibodies, the sections were first blocked with blocking buffer containing 10% goat serum diluted in 3% BSA-PBS for 1hour at room temperature. The sections were then incubated for 1hour with an antibody cocktail containing the following antibodies: F4/80, CD206 and PDPN; or B220 and CD3 (see **Supplementary Table 2**). Washed sections were incubated for 30 minutes in the dark with a cocktail containing the secondary antibodies (see **Supplementary Table 2**) and Hoechst 34580 (H21486, Invitrogen by Thermo Fisher Scientific), which was used for nuclear staining. The slides were mounted using Prolong Gold Antifade reagent (Invitrogen by Thermo Fisher Scientific), then left to dry in the dark for 20hours and sealed with nail polish.

#### New Zealand black and white

Salivary glands from NZBW-F1 mice (n=7) were used as a spontaneous model of TLS in autoimmune disease development. Salivary gland tissues were fixed in 10%NBF and embedded in paraffin. Indirect detection by fluorescence was based on the Opal Multiplex IHC method (Akoya Biosciences) and was performed on 4µm thick sections, with some modifications to the original protocol from the manufacturer. See **Supplementary Table 3** for details regarding the order of labelling antibodies and their corresponding detection system and the OPAL fluorophore applied. The sections were first deparaffinized and rehydrated using xylene, followed by decreasing concentrations of ethanol before washing in deionized water. Subsequently, a secondary fixation step was performed with 10%NBF for 20miutes. Epitope retrieval was performed using a citrate buffer. Endogenous peroxidases were blocked with 3% hydrogen peroxide, then anti-PDPN antibody was applied, followed by Polink-2 Plus HRP Anti-Syrian hamster DAB detection kit (D86-18, Golden Bridge International) and signal amplification using Opal Fluorophore. Antibody stripping was performed via microwave treatment with citrate buffer, followed by blocking for nonspecific protein binding using a detection system with 10% normal goat serum in PBS. This process was repeated for the additional antibodies (F4/80, CD206 and CX3CR1), followed by the application of the Envision+ system-HRP/DAB anti-rabbit detection kit (K4011, Dako), signal amplification with different OPAL Fluorophores and antibody stripping. The final steps involved the application of TSA-DIG and Opal Polaris 780 (SKU FP1501001KT, Akoya Biosciences) for signal amplification, with no subsequent microwave treatment. The sections were then counterstained with DAPI (SKU FP1490, Akoya Biosciences), mounted with Prolong Gold antifade mounting medium (P36930, Invitrogen by Thermo Fisher Scientific) and sealed with nail polish prior to image acquisition and analysis.

### Image acquisition and analysis

Images of both the fluorescence-labeled and H&E-stained sections were acquired using a VS120 Virtual Slide Microscope (Olympus) at 20X magnification. Analysis of the fluorescence labelled images was performed using QuPath software V-0.5.1 (23). Automatic cell detection was performed using Hoechst nuclear staining. Cell detection was followed by creating a cell classifier for each marker channel according to whether they were positive for the marker. The cell classifier was set by combining the pixel intensity threshold and machine learning for marker classification. In the end the classifiers were combined and applied to the detected cells. See **Supplementary Figure 1** for the workflow pipeline for the analysis. This analysis was performed on whole tissue sections, and three different regions of interest (ROIs) in the tissue section were created to compare changes within the tissue. ROI1 – Region with low inflammation, ROI2 – Region with inflammation, and ROI3 – Region with T and B cell aggregation. The locations of the different ROIs were defined based on sections with signs of high inflammation burden, the day 8 and day 16 samples. In these samples, tertiary lymphoid structures (TLSs) were located in close proximity to intercalated ducts and lymphatic vessels. For samples with low degree of inflammation and no TLSs present, the ROIs were placed in similar locations in the tissue due to comparisons between the different section. Statistical analyses were performed using GraphPad Prism 7.05. One-way analysis of variance (ANOVA) with Dunnett’s multiple comparison test was used to test the values against day 0. To test for statistical significance in ROIs, two-way ANOVA with Dunnett’s and Tukey’s multiple comparisons tests were applied to test for significance against day 0 and to test for significance between the different ROIs.

### RNA isolation, cDNA synthesis and real time PCR

RNA was extracted from OCT-embedded salivary gland tissue using a column-based approach. First approximately 200µm of tissue was cut with a cryostat, placed in 2mL MagNA Lyser Green Beads tube (3358941001, Roche) containing 600µL TriReagent (Zymo Research) and stored on dry ice. The samples were then homogenized in a homogenizer “Precelys 24” at 5000rpm for 30 seconds, before being transferred to marked 1.5mL DNA-LoBind Eppendorf tubes. RNA extraction was performed using the Direct-zol™ RNA MiniPrep kit (R2051, Zymo Research) and the necessary steps were followed according to the manufacturer’s protocol. The extracted RNA was eluted in 25µl RNASe-free dH_2_O and the RNA concentration and purity were determined using a NanoDrop 2000 spectrophotometer (Thermo Fisher Scientific, US). The RNA integrity number (RIN value) of the isolated RNA was assessed using the Agilent RNA 6000 Nano Assay protocol on the Agilent 2100 Bioanalyzer. A High-Capacity cDNA Reverse Transcription kit (4368813, Applied Biosystems^®^ by Life Technologies, US) was used to synthesize cDNA, 500ng of RNA was used in a 40µL reaction. Real-time qPCR was performed with a LightCycler^®^ Analyzing machine (Roche Holding AG) using TaqMan gene expression assays (Thermo Fisher Scientific). An overview of the gene expression assays is provided in **Supplementary Table 4**. TaqMan Fast Universal PCR master mix (2X) and gene expression assays were all obtained from Applied Biosystems (Foster City, USA). The reference gene *TBP* for each mouse was used to normalize gene expression. The average of the control mice served as a reference for fold change, which was calculated using the ΔΔCt method (24). One-way ANOVA with the *post-hoc* analysis Dunnett’s multiple comparison test was performed to test for differences between populations using GraphPad Prism 7.05 software.

## Results

### T and B cell aggregates were induced in the salivary glands of LucAdV5 infected mice in the virus-induced primary Sjögren’s disease mouse model

**Figure 1:**
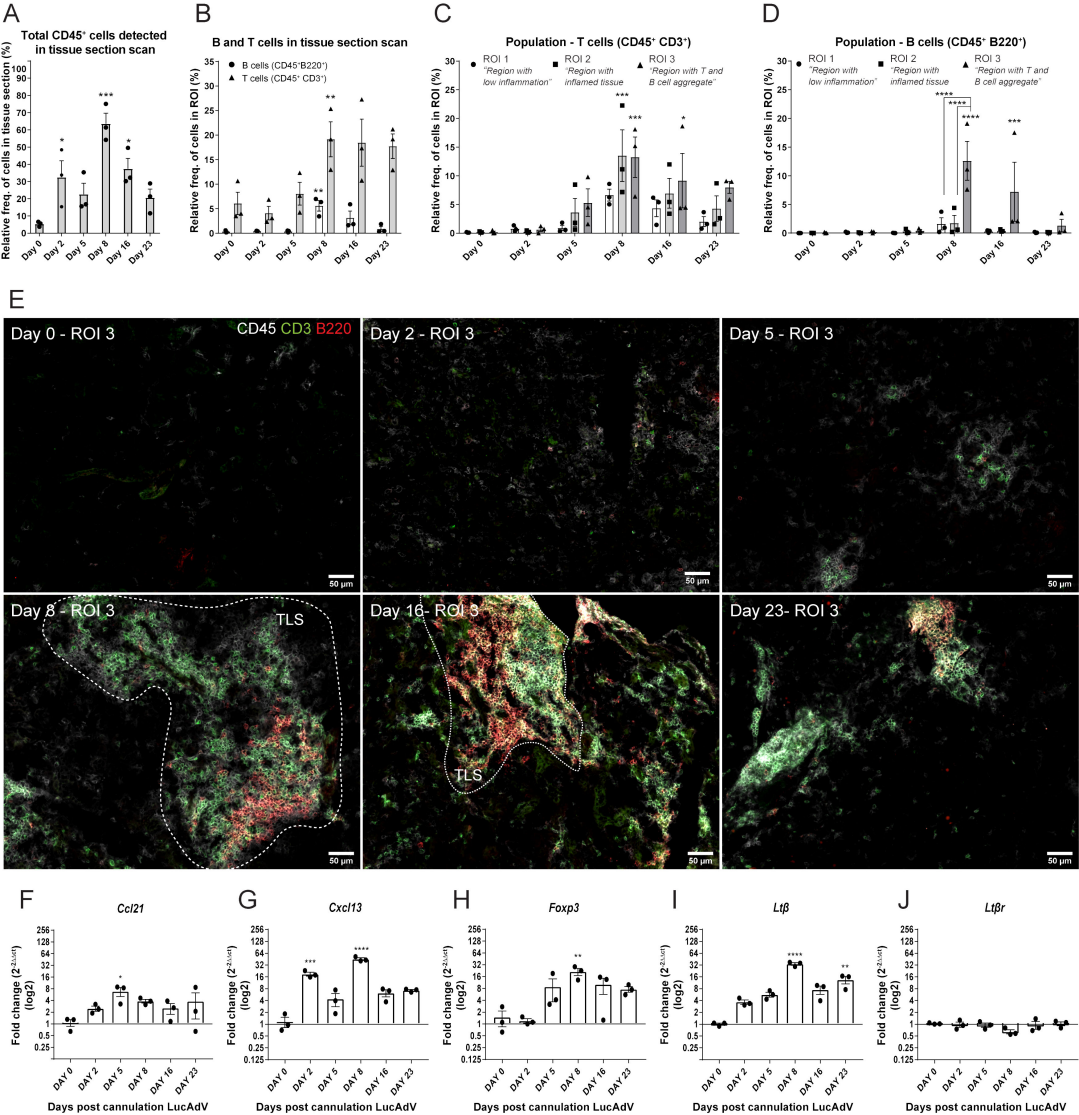
TLS development in LucAdV5-induced primary Sjögren’s disease model. Aggregation of T and B cells during LucAdV5-induced primary Sjögren’s disease was used to assess the development of TLSs in the salivary glands of C57BL/6J mouse model. Immunofluorescence (IF) labelling of CD45, CD3 and B220 was performed on salivary gland cryosections at six different time points during LucAdV5 infection. **(A)** Relative frequency of CD45^+^ cells in scanned tissue sections, based on the total number of cells detected in the tissue. **(B)** Relative frequency of B cells (CD45^+^B220^+^) and T cells (CD45^+^CD3^+^) based on the number of infiltrated CD45^+^ cells in the tissue section. **(C)** Relative frequencies of T cells and **(D)** B cells in three regions of interest. **(E)** Representative images of ROI 3; B and T cell aggregation for different time points during inflammation processes [CD45(white), CD3(green) and B220(red)]. Changes in gene expression of **(F)** *Ccl21*, **(G)** *Cxcl13*, **(H)** *Foxp3*, **(I)** *Ltβ* and **(J)** *Ltβr*are shown as fold changes. Data are presented as mean ± SEM. One-way ANOVA with the *post-hoc* analysis Dunnett’s multiple comparison test was used for panels **(A, B, F–J)**, while two-way ANOVA statistical test with the *post-hoc* analysis and Tukey’s multiple comparisons tests were applied for panels **(C, E)** * equals a p-value <0.05, ** equals a p-value <0.01, *** equals a p-value <0.001, **** equals a p-value <0.0001. Non-statistically significant values are not shown. The statistics of the IF-stained tissue is based on cell segmentation and fluorescence classification performed with QuPath V-0.5.1.

We studied the establishment of TLSs in the salivary glands of LucAdV5 infected mice using an inducible mouse model of primary Sjögren’s disease. Representative salivary gland tissue images of immune cell infiltration during infection-induced inflammation are presented in **Supplementary Figure 1A**. The relative frequency of CD45^+^ cells in the salivary gland tissues increased during LucAdV5 infection, with a peak observed at day 8 post-infection (**Figure 1A**). T cells (CD45^+^CD3^+^) infiltrated the salivary glands on day 5, with a statistically significant peak observed on day 8 post-LucAdV5 infection. Infiltrating B cells (CD45^+^B220^+^) also showed a statistically significant peak on day 8 post-infection (**Figure 1B**). The stages of T and B cell aggregation were monitored at different time points and the relative frequencies of T and B cells in the selected ROIs were calculated (**Figures 1C, D**). Comparing the three different ROIs, the ROI1 – Region with low inflammation, the ROI2 – Region with inflammation, and the ROI3 – Region with T and B cell aggregation, revealed that T cells were found in both ROI2 and ROI3 (**Figure 1E**). B cells were only found clustered in ROI3, always together with T cells both at day 8 and day 16 post-infection (**Figure 1D**). Thus, the main difference between T and B cell aggregation is the organization of the initial T/B cell compartmentalization to the segregation of T and B cells in separate areas (**Figures 1C–E**).

To confirm the observations of T and B cell staining, mRNA gene expression studies were performed. The gene expression of the chemokines involved in T cell and B cell recruitment, *Ccl21* (**Figure 1F**) and *Cxcl13* (**Figure 1G**) were both significantly increased in the salivary glands during LucAdV5 infection compared to uninfected tissue. *Ccl21* mRNA expression peaked on day 5 when T cells were observed in the tissue. The expression of *Cxcl13* mRNA was increased in salivary gland tissues, with two main statistically significant peaks. The first peak occurred during the acute phase of infection on day 2 post-infection. The second main peak was observed on day 8, at the time point when TLS development was initiated, and infiltrating B cells were observed. A significant increase in mRNA expression was observed for *Foxp3* (**Figure 1H**) and *Ltb* (**Figure 1I**) on day 8, coinciding with T and B cell infiltration into the tissue. The expression of *Ltβ* mRNA was significantly elevated in the tissue on day 8 and day 23 post LuCAdV5 infection. The gene expression of the LTβ receptor, *Ltβr*, was stable during LuCAdV5 infection and no statistically significant changes in expression were observed during the stages of infection-induced inflammation (**Figure 1J**).

### The myeloid cell population revealed an F4/80^+^ macrophage population expressing CX3CR1 and PDPN

**Figure 2:**
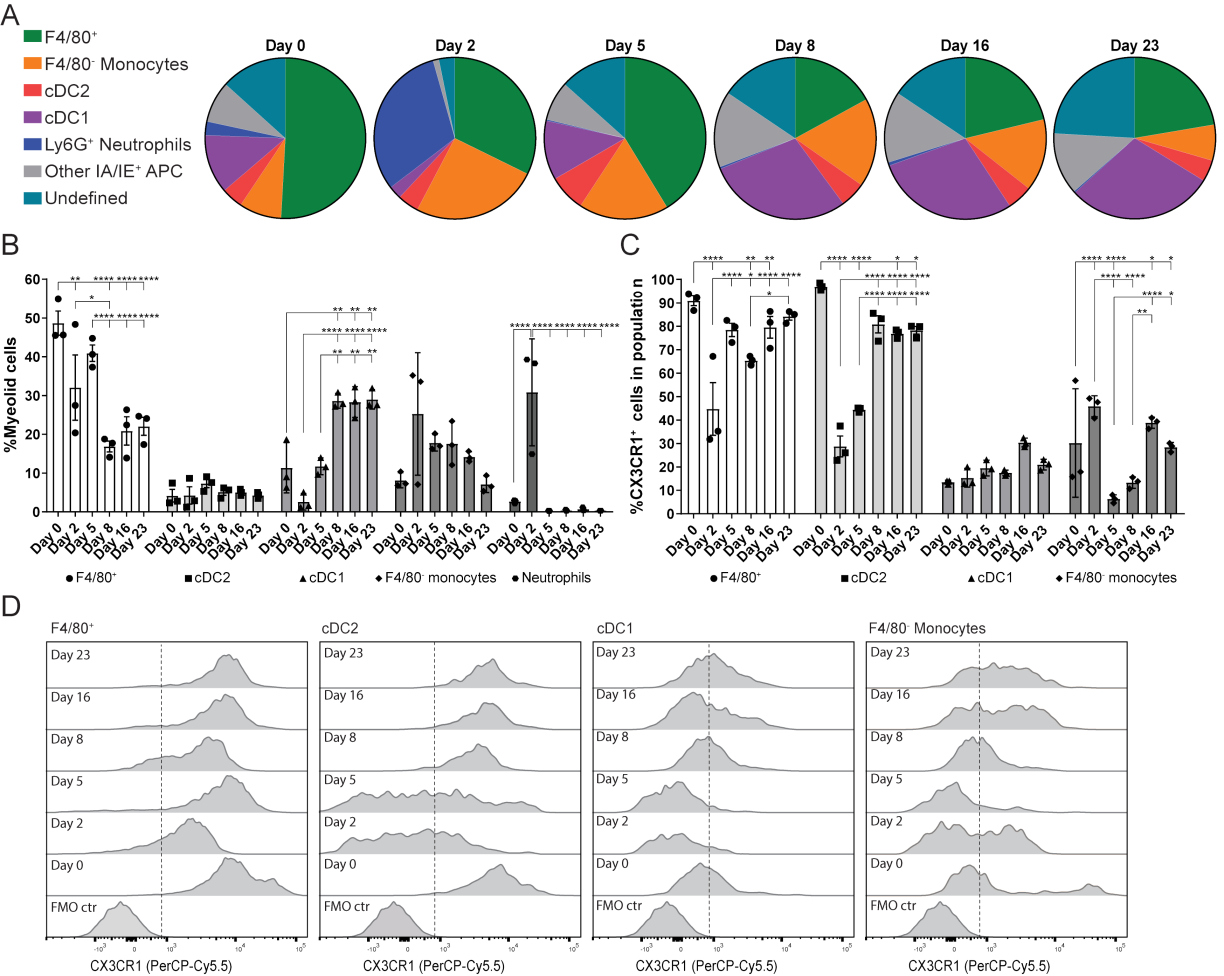
Myeloid cell population response and dynamics in LucAdV5-induced inflammation with focus on the expression of CX3CR1. **(A)** Pie chart presentation of flow cytometric analyses showing the changes in myeloid cell populations studied in the submandibular salivary glands from before (day 0) and after (day 2, 5, 8, 16 and 23) infection of LucAdV5 in C57BL/6J mice. **(B)** Changes in the F4/80^+^ macrophage, CD11b^+^ DC2, CD11c^+^ DC1, F4/80^-^ monocyte and neutrophil populations represented as the relative frequency of the myeloid cell population. **(C)** Relative frequencies of CX3CR1 positive cells in the populations of F4/80^+^ macrophages, CD11b^+^ cDC2, CD11c^+^ cDC1 and F4/80^-^ monocytes. **(D)** Representative histogram of CX3CR1 expression in F4/80^+^ macrophage, cDC2, cDC1 and F4/80^-^ monocyte populations. The expressions are shown as a percentage of the maximum count and the dashed line indicates the cut-off between positive and negative events. The data are presented as mean with +/- SEM. One-way ANOVA and *post-hoc* analysis Dunnett’s multiple comparison test was performed to test for differences between the populations prior to LucAdV5 infection (day 0) and the populations after LucAdV5 infection (day 2, 5, 8, 16 and 23). Statistically significant values are marked with “*”, where: * equals a p-value <0.05, ** equals a p-value <0.01, *** equals a p-value <0.001, **** equals a p-value <0.0001. Non-statistically significant values are not shown in the figures. The gating strategy of myeloid cell populations in the salivary glands is shown in **Supplementary Figure 2**.

The next aim of this study was to determine the dynamics of the myeloid cell population during LucAdV5 induced inflammation. The focus was on the F4/80^+^Ly6G^-^ cell population, which was defined as the F4/80^+^ macrophages in this study. Flow cytometric analysis of the myeloid cell population revealed a dynamic pattern, which corresponded to the different phases of LucAdV5 induced inflammation processes (**Figures 2A, B**). The gating strategy of the myeloid cell population can be found in **Supplementary Figure 2A**.

In the uninfected salivary gland tissue, the myeloid cell population was predominately dominated by the F4/80^+^ macrophage population (48.7± 5.4%). However, in the acute phase of the inflammation at day 2 post LucAdV5 infection, an influx of Ly6G^+^ neutrophils (30.8± 13.8%) and F4/80^-^ monocytes (25.3 ± 15.8%) was detected in the tissue, while a decrease in the F4/80^+^ macrophage population (32.0 ± 14.6%) and CD11c^+^CD11b^-^ cDC1 (2.6 ± 2.2%) were observed (**Figure 2B**). In the transition phase between acute phase inflammation and the phase where TLS initiation occurs, on day 5 post-infection, the macrophage population slightly increased towards a steady state, together with an increase in cDC1 populations. Simultaneously, compared with the acute phase, the disappearance of neutrophils (0.3 ± 0.06%) and a decrease in the F4/80^-^ monocyte population (17.8 ± 2.1%) are observed (**Figures 2A, B**). The cDC1 population remained one of the main myeloid cell populations throughout the stages of LucAdV5 infection and significantly increased from day 8 post-infection. The tissue expression of CD11c^+^CD11b^+^ cDC2 myeloid cell population was generally stable showing no major response to LucAdV5 induced inflammation (**Figure 2B**).

CX3CR1 expression in myeloid cell populations has been linked to TLS involvement in the gut (16). Unbiased t-distributed stochastic neighbor embedding (t-SNE) analysis combined with conventional gating of the myeloid cell population revealed that CX3CR1 was predominantly expressed in the F4/80^+^ macrophage and cDC2 populations during different stages of LucAdV5 infection (**Supplementary Figures 2B, C**). Further analysis of CX3CR1 expression among F4/80^+^ macrophages, F4/80^-^ monocytes, cDC2, and cDC1 myeloid cell populations confirmed the trends observed in the tSNE plots (**Figures 2C, D**). Statistical analysis of CX3CR1 revealed that most of the F4/80^+^ macrophages expressed this marker (**Figure 2D**), where median fluorescence intensity (MFI) of the CX3CR1 expression was relatively high (**Figure 2D**). However, on days 2 and 8 post-infection, a significant decrease in the MFI and the relative frequency of CX3CR1^+^ cells within the F4/80^+^ macrophage population decreased, from 90 ± 3.69% in uninfected tissue to 44.74 ± 19.53% at day 2 post-infection and 65.39 ± 1.92% at day 8 (**Figure 2C**). After these drops, the relative frequencies of CX3CR1^+^ cells among the F4/80^+^ macrophages increased to the state before infection. At days 2 and 5 post-infection, a similar trend was observed among the cDC2 population (**Figure 2C**). In addition, these cells had a relatively high CX3CR1 expression (MFI), similar to the F4/80^+^ macrophages (**Figure 2D**). In F4/80^+^ macrophages and cDC2, CX3CR1 expression significantly decreased throughout the inflammation process. Only a small portion of both cDC1 and F4/80 monocyte populations had a CX3CR1 expression phenotype in uninfected salivary gland tissue. However, the cDC1 population showed a small statistically significant increase in expression at day 5, 16 and 23 post-infections. Monocytes can be characterized by varying expression of CX3CR1. A significant increase in the relative frequency of CX3XR1^+^ monocytes in the acute phase on day 2 post-infection was observed (**Figure 2C**) before it dropped on days 5-8 (**Figures 2C, D**), and increased again on day 16 post-infection.

**Figure 3:**
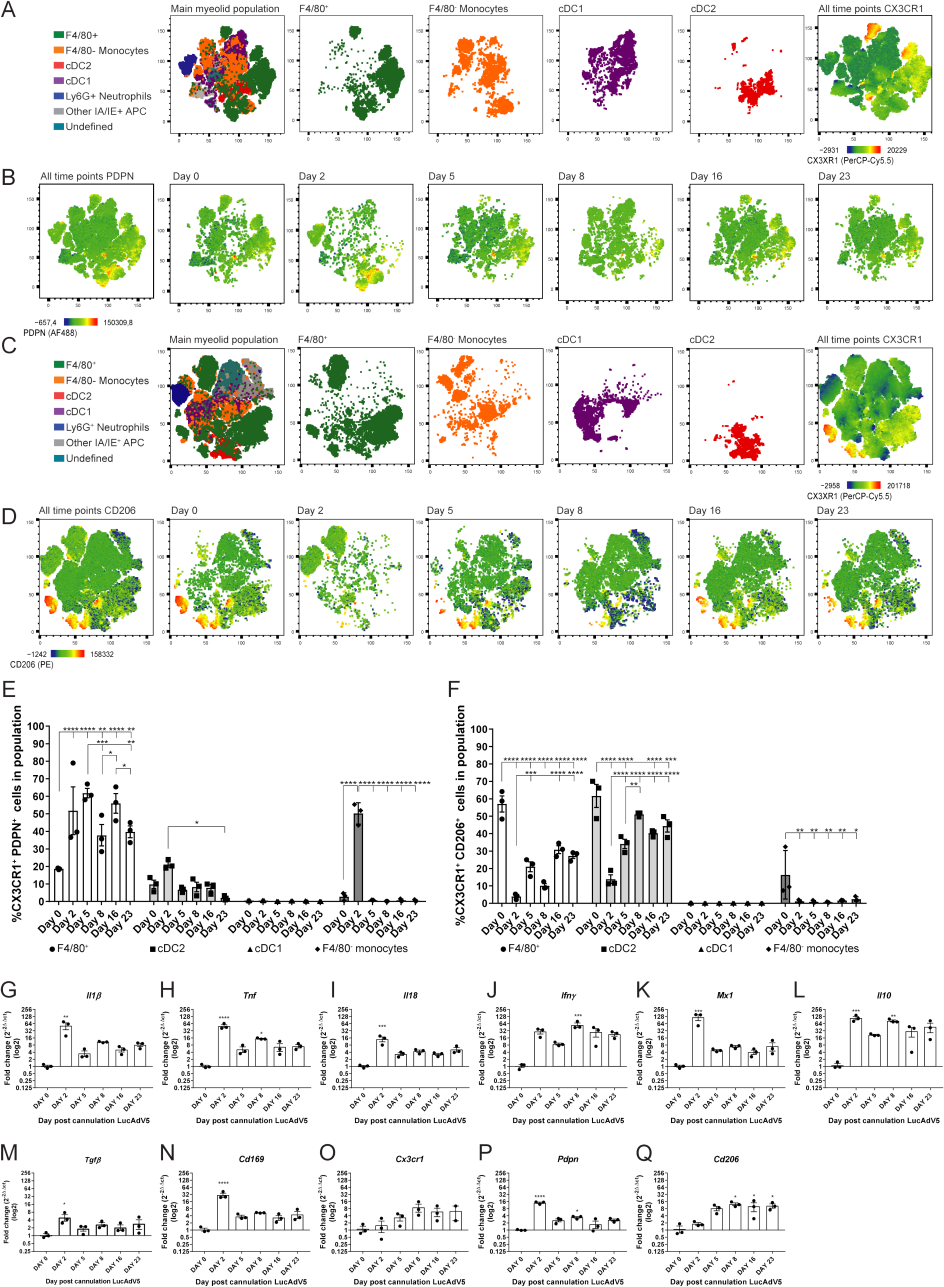
CX3CR1 expressing myeloid cells and their PDPN and CD206 phenotypes. Myeloid cell populations were gated into the tSNE plot analysis for the two panel setups of the flow cytometry experiments. **(A)** Myeloid cell clustering for panel 1, with emphasis on F4/80^+^ macrophages, F4/80^-^ monocytes, cDC1 and cDC2. Heatmap visualization of CX3CR1 expression. **(B)** Heatmap visualization of PDPN expression during LucAdV5 infection. **(C)** Myeloid cell clustering for panel 2 and panel 1 with an emphasis on F4/80^+^ macrophages, F4/80^-^ monocytes, cDC1 and cDC2. Heatmap visualization of CX3CR1 expression. **(D)** Heatmap visualization of CD206 expression during LucAdV5 infection. Relative frequencies of **(E)** CX3CR1^+^PDPN^+^ and **(F)** CX3CR1^+^CD206^+^ cells in the populations of F4/80^+^ macrophages, CD11b+ cDC2, CD11c+ cDC1 and F4/80^-^ monocytes. Changes in gene expression of **(G)** *Il1β*, **(H)** *Tnf*, **(I)** *Il18*, **(J)** *Ifny*, **(K)** *Mx1(Ifnα)*, **(L)** *Il10*, **(M)** *Tgfβ*, **(N)** *Cd169*, **(O)** *CX3CR1*, **(P)** *Pdpn* and **(Q)** *Cd206* are shown as fold changes normalized to uninfected salivary gland tissue. The data are presented as mean with +/- SEM. One-way ANOVA and *post-hoc* analysis Dunnett’s multiple comparison test was performed to test for differences between the populations prior to LucAdV5 infection (day 0) and the populations after LucAdV5 infection (day 2, 5, 8, 16 and 23). Statistically significant values are marked with “*”, where: * equals a p-value <0.05, ** equals a p-value <0.01, *** equals a p-value <0.001, **** equals a p-value <0.0001. Non-statistically significant values are not shown in the figures.

PDPN expression among the selected myeloid cell populations showed that several of them had increased expression in the acute phase of inflammation on day 2 post-infection (**Figures 3A, B, E**). The combination of CX3CR1 and PDPN in the myeloid population revealed that the F4/80^+^ macrophage population showed a significant increase in these two markers throughout the infection phases (**Figure 3E**). The increase in the CX3CR1^+^PDPN^+^ F4/80^+^ macrophage population had statistically significant peaks at day 2, 5 and 16 post LucAdv5 infection (**Figure 3E**). These timepoints correlate with the acute phase and TLS developmental phase in inflammatory processes. The cDC2 population included a small subpopulation of CX3CR1^+^PDPN^+^ cells, with a small statistically significant decrease from day 2 post-infection to day 23 post-infection. The incoming monocytes showed a significantly high expression of both CX3CR1 and PDPN on day 2 post-infection (**Figure 3E**).

The mannose receptor CD206 was found in the F4/80^+^ macrophages, cDC2 and F4/80^-^ monocyte myeloid cell population during LucAdV5 infection, but not in cDC1 population (**Figures 3C, D, F**). The CX3CR1^+^F4/80^+^ macrophage population showed a decrease in the relative frequency of CD206^+^ cells compared to the uninfected tissue at day 0 with the lowest expression at day 2, like the CX3CR1^+^cDC2 population (**Figure 3F**). Some CX3CR1^+^ F4/80^-^ monocytes showed a CD206 expression phenotype in the uninfected salivary gland tissue. In the infected tissue, CD206 expression was drastically decreased, and the phenotype was no longer observed.

Further analysis of the total mRNA expression of the cytokine pattern in salivary gland tissue confirmed the observation of the myeloid cell population. mRNA levels of *Il1β* (**Figure 3G**)*, Tnf* (**Figure 3H**) and *Il18* (**Figure 3I**) were significantly increased in the acute phase of the infection compared to those in uninfected salivary gland tissue. In addition, *Tnf* showed a statistically significant peak at day 8 post-infection, corresponding with in the early TLS development phase. The mRNA expression of *Ifny* (**Figure 3J**) increased throughout the inflammation processes, with a statistically significant peak at day 8 post-infection. The response marker of the interferon (IFN)-*α* response, *Mx1* (**Figure 3K**), revealed an early response with a main, statistically significant peak at day 2 post-infection. The immunoregulatory chemokines *Il10* and *Tgfβ s*howed an increase in their mRNA expression throughout the inflammatory processes. *Il10* mRNA revealed statistically significant peaks at day 2 and 8 post-infection, while the mRNA expression of *Tgfβ* had one main significant peak in the acute phase of inflammation on day 2 post-infection (**Figures 3L, M**). The mRNA of the *Cd169* gene, which is associated with macrophages in lymphoid tissues (25), was increased through LucAdV5 induced inflammation with a significant peak at day 2 post-infection (**Figure 3N**).

Next, we looked at the mRNA expression of genes related to results from the flow cytometry analysis; *Cx3cr1* (**Figure 3O**), *Pdpn* (**Figure 3P**), and *Cd206* (**Figure 3Q**) mRNAs. *Cx3cr1* mRNA showed an increased expression in the LucADV5 induced inflammation processes, but no statistically significant peaks were detected. *Pdpn* mRNA showed statistically significant expression in the acute phase and in the early TLS developing phase at day 2 and 8 post-infection, respectively. The gene for the mannose receptor *Cd206* showed an expression that increased in the later stages of infection, from the TLS developmental phase to the resolution phase, with significant peaks at day 8, 16 and 23 post-infection.

### We observed F4/80^+^PDPN^+^CD206^+^ macrophages in the salivary gland tissue, and F4/80^+^ macrophages surrounding the TLSs at day 16 post LucAdv5 infection

**Figure 4:**
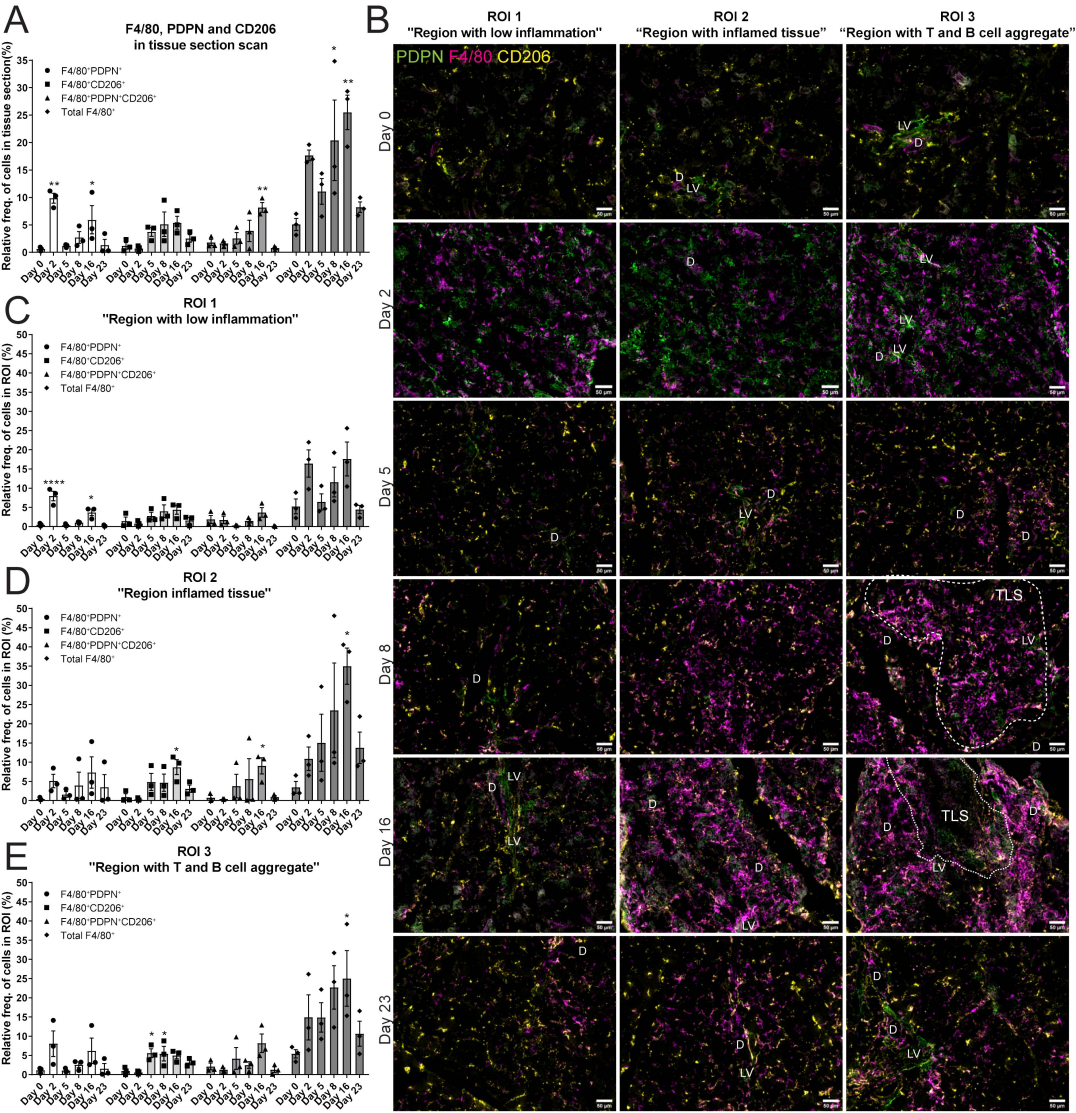
Localization of F4/80^+^, PDPN^+^ and CD206^+^ cells in salivary gland tissue during LucAdv5 infection. Immunofluorescence labelling of F480, PDPN and CD206 expressing cells was performed on salivary gland cryosections at six different time points during LucAdV5 infection. **(A)** Relative frequencies of F4/80^+^PDPN^+^, F4/80^+^CD206^+^, F4/80^+^CD206^+^ PDPN^+^ and total F4/80^+^ cells in the scanned tissue section, based on the total number of cells detected in the tissue. **(B)** Representative images showing F4/80 (magenta), PDPN (green) and CD206 (yellow) labelling of the three regions of interest (ROIs) at different time points. The relative frequency of the F4/80 labelling profiles in **(C)** Region 1 “region with low inflammation”, **(D)** ROI 2 “Region with inflamed tissue”, **(E)** ROI 3 “Region with T and B cell aggregate”. Abbreviations salivary gland ducts **(D)**, lymphatic vessel (LV) and tertiary lymphoid structure (TLS). Data presented as mean +/- SEM. Two way ANOVA and the *post-hoc* analysis Dunnett’s multiple comparison test were performed to test for differences between the populations prior to LucAdV5 infection (day 0) to the populations after LucAdV5 infection (day 2, 5, 8, 16 and 23. Statistically significant values are marked with “*”, where: * equals a p-value <0.05, ** equals a p-value <0.01, **** equals a p-value <0.0001. Non-statistically significant values were not included in the graphs. Images of the scanned IF-stained sections are shown in **Supplementary Figure 1B**. The statistics of IF-stained tissue were based on cell segmentation and fluorescence classification performed using QuPath V-0.5.1.

The next task in this study was to reveal the expression of F4/80^+^ macrophages in salivary gland tissues. To address this, immunofluorescence staining for F4/80, PDPN and CD206 was performed (**Supplementary Figure 1B**). The relative frequency of the total F4/80^+^ cell population significantly increased during LucAdV5 infection, with peaks at day 8 and day 16 post-infection (**Figure 4A**). Cells expressing both F4/80 and PDPN in the tissue showed two main peaks, one peak in the acute phase of inflammation on day 2, and one peak on day 16, concurrently with TLS formation in the salivary glands. The relative frequency of F4/80^+^ CD206^+^ cells increased in the tissue at the later inflammation stages from day 5 to day 16. Interestingly, a population of F4/80^+^ cells expressing both PDPN and CD206 was detected in the normal tissue and increased during inflammation with a significant peak at day 16 post-infection. Comparing the relative frequency of F4/80^+^ cell populations in the different regions, defined by B and T cell staining, could pinpoint the expression of the macrophages in the tissue during the inflammation process in response to LucAdV5 infection (**Figure 4B**). As expected, there was no major statistically significant increase in the number of F4/80^+^ cells detected in ROI 1 (Region with low inflammation) during the LucAdV5 infection (**Figure 4C**). However, statistically significant increases and peaks in F4/80^+^PDPN^+^ cells were observed in this region at day 2 (7.96 ± 2.15%), and day 16 (3.70 ± 1.49%) post-infection. In ROI 2 - Region with inflamed tissue (**Figure 4D**), a major increase in the total number of F4/80^+^ cells was detected with peaks at day 16 post-infection (34.99 ± 4.71%). The F4/80^+^CD206^+^ cell population increased in this region, with a peak at day 16 post-infection (8.69 ± 1.94%). It was also possible to detect some F4/80^+^PDPN^+^CD206^+^ cells in this region, which showed an increasing expression until TLS development on day 16. In the last region, ROI 3 – Region with T and B cell aggregation (**Figure 4E**), the organization of F4/80^+^ macrophages changed from merging with the lymphocytes on day 8 to encapsulating the TLSs on day 16 after LucAdv5 infection (**Figure 4B**). F4/80^+^PDPN^+^ and F4/80^+^CD206^+^PDPN^+^ cells were detected in this region at day 16, surrounding the TLSs. F4/80^+^CD206^+^ cells were predominantly detected in the early phase of TLS development at day 5 and 8 post LucAdV5 infection. Overview images of the regions with segmentation are presented in **Supplementary Figure 1C**.

### Localization of F4/80^+^, PDPN^+^, and CD206^+^ cells in the salivary gland tissue of NZBW-F1 mice revealed that F4/80^+^PDPN^+^ macrophages are surrounding the TLSs

**Figure 5:**
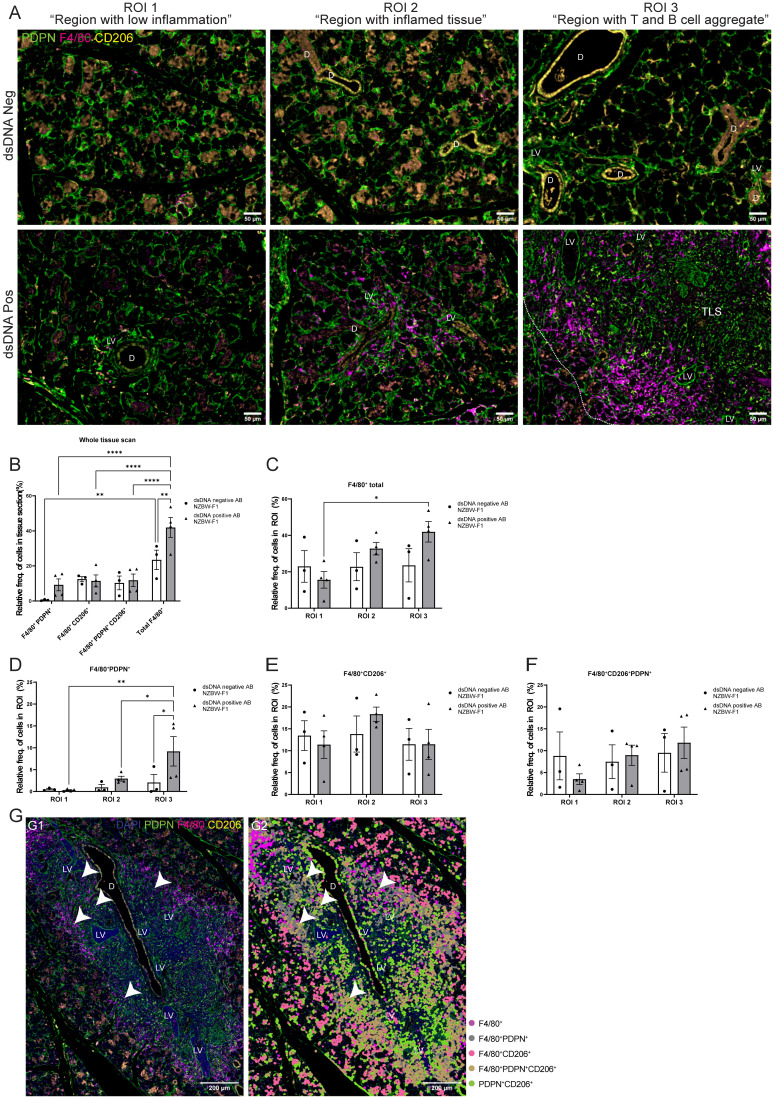
Organization of the F4/80^+^ macrophages in salivary gland tissue from NZBW-F1 mice with focus on their expression of PDPN and CD206. Immunofluorescence labelling of F480, PDPN, and CD206 expressing cells was performed on paraformaldehyde-fixed and paraffin-embedded salivary gland tissue from NZBW-F1 mice negative or positive for dsDNA autoantibodies, which are clinical markers of SLE. **(A)** Representative images of F4/80 (magenta), PDPN (green) and CD206 (yellow) labelling of the three different regions of interest (ROIs) in the salivary glands of NZBW-F1 mice. **(B)** Relative frequency of F4/80^+^PDPN^+^, F4/80^+^CD206^+^, F4/80^+^CD206^+^PDPN^+^ and total F4/80^+^ cells in whole scanned tissue section. Relative frequency of **(C)** F4/80^+^ cells, **(D)** F4/80^+^PDPN^+^, **(E)** 4/80^+^CD206^+^ and **(F)** F4/80^+^CD206^+^PDPN^+^ in the three different regions, ROI1 1 “region with low inflammation”, ROI 2 “Region with inflamed tissue”, and ROI 3 “Region with T and B cell aggregate”. **(G)** Representative image of salivary gland TLSs with (G2) and without (G1) overlay of the cell segmentation. Unclassified cells and cells marked only with CD206 or PDPN were excluded from the figure. White arrowheads points to F4/80^+^CD206^+^PDPN^+^ macrophages located within the TLS. Abbreviations salivary gland ducts **(D)**, lymphatic vessel (LV) and tertiary lymphoid structure (TLS). Data presented as mean +/- SEM. Two-way ANOVA and *post-hoc* Tuckey’s multiple comparison tests were performed to test for differences. Statistically significant values are marked with “*”, where: * equals a p-value <0.05, ** equals a p-value <0.01, **** equals a p-value <0.0001. Non-statistically significant values are not included in the graphs. The statistics of IF-stained tissue were based on cell segmentation and fluorescence classification performed using QuPath V-0.5.1.

After studying the organization of macrophages in the LucAdV5 infected mouse model, we wanted to compare the results with the expression and organization of macrophages in the salivary glands of NZBW-F1 mice. NZBW-F1 mice were grouped based on the presence or absence of dsDNA autoantibodies (dsDNA-positive or dsDNA-negative), which serve as clinical markers of SLE. We utilized IF labelling to identify F4/80, PDPN, and CD206 expressing cells within salivary gland tissues (**Figure 5A**; **Supplementary Figure 3**).

Next, we compared the macrophage populations in the whole tissue sections (**Figure 5B**) and across three ROIs (**Figures 5C–F**) in the salivary glands. This provided visual evidence of the distribution and co-localization of these markers in the tissue, in addition to quantitative data of the relative frequency of macrophages expressing combinations of F4/80, PDPN, and CD206 (**Figures 5B–F**). The relative frequency of the total F4/80^+^ cell population in the salivary gland sections from dsDNA-positive mice (41.94 ± 11.32%) was higher than that in dsDNA-negative mice (23.46 ± 9.59%) (**Figure 5B**). Comparing the frequency of F4/80^+^ macrophages in the different ROIs confirmed this observation (**Figure 5C**). Then, we focused on the localization of macrophages labelled with PDPN and CD206. The PDPN^+^F4/80^+^ labelled macrophages were increased in the regions containing a TLS, ROI 3 “region with T and B cell aggregates”, when compared to the different regions within the sections of dsDNA-positive mice, and with the regions of the dsDNA-negative mice (**Figure 5D**). Indicating, macrophages that are in region with TLSs show increased expression of PDPN. There were no major differences in the relative frequencies of CD206^+^ and CD206^+^PDPN^+^ macrophages in the three different regions between dsDNA-negative and dsDNA-positive NZBW-F1mice. Comparing original image (**Figure 5**G–1) with an overlay (**Figure 5**G–2) of the cell segmentation on the images with TLSs revealed how the organization of the F4/80^+^ macrophages encapsulated the TLSs (**Figure 5G**). Here, we observed that the majority of F4/80^+^macrophages express PDPN, and interestingly F4/80^+^PDPN^+^CD206^+^ macrophages seem to share a similar organization. We also observed a few F4/80^+^PDPN^+^CD206^+^ macrophages inside the TLSs (**Figure 5G**, white arrows). The F4/80^+^CD206^+^ macrophages were predominantly located outside the TLS periphery of the tissue.

## Discussion

In this study, we evaluated the dynamics and changes in the phenotype of salivary gland macrophages during inflammation and lymphoid neogenesis induced by LucAdV5 infection. Here, we noticed different phases of lymphoid organization from the acute phase to the resolution phase of infection induced inflammation. We observed chemokines for T cell recruitment and the presence of CD3^+^ T cells in the tissue as early signs of TLS formation, whereas B cell infiltration was observed in the aggregation phase of TLS development and maturation. Comparing the virus-induced LucAdV5 mouse model with the spontaneous NZBW-F1 mouse model of human SLE and Sjögren’s disease (20, 21), we describe the involvement of macrophages in pathogenesis of these two autoimmune diseases with focus on their expression of PDPN. Infiltration of immune cells and organization of TLSs in salivary gland tissue are hallmarks of Sjögren’s disease, which can lead to an increased risk of lymphoma and systemic manifestations (26, 27).

TLS neogenesis is an important effector of the pathogenicity of Sjögren’s disease, which is often associated with a high focus score in salivary gland biopsies and is a marker for deteriorating disease prognosis (27–30). The development of TLSs has also been associated with an increased risk of developing non-Hodgkin’s lymphoma in the affected salivary glands, with local production of autoantibodies and clonal B-cell expansion (6, 31). While the development of TLSs in Sjögren’s disease is a critical marker of disease progression, the cellular constituents within these structures orchestrate the intricate balance between proinflammatory and regulatory mechanisms (32, 33). Among these, macrophages have emerged as pivotal players, not only because of their abundance, but also because of their functional diversity (2). The identification of two distinct F4/80^+^ macrophage populations, CX3CR1^+^PDPN^+^ and CX3CR1^+^CD206^+^, within inflamed salivary glands offers new insights into the heterogeneity of the macrophage response in Sjögren’s disease. Here, both tissue-resident and monocyte-derived macrophages can contribute to several processes of initiation and maintenance of TLS development and function.

In our model, we observed that the genes responsible for proinflammatory cytokines were increased during the acute phase of inflammation. Thus, macrophages may be involved in immune cell recruitment through the production of proinflammatory cytokines, such as IL1β and TNF, together with tissue stromal cells and endothelial cells (2, 34, 35). The presence of the proinflammatory cytokine IL-18 and active inflammasomes are often associated with disease onset and progression in both patients and mouse models of Sjögren’s disease (35–38). One of the main producers of IL-18 are macrophages. IL-18 is important to stimulate proliferation and IFNγ signaling among naïve T cells, CD8+ and NK cells (39). We observed increased *IL-18* gene expression in the salivary gland tissue of LucAdV5 induced inflammation, indicating that salivary gland macrophages are important mediators of proinflammatory processes and in the recruitment and stimulation of lymphocytes.

The recruitment of B cells is dependent on the chemokine CXCL13, which is necessary for the development of TLSs (2, 40, 41). In one of the early studies on CXCL13 expression in the developing lymphoid tissues of patients with rheumatoid arthritis (RA) and ulcerative colitis (UC), macrophages and activated monocytes were found to be the main sources of CXCL13 in the tissues where TLSs were formed (42). These findings are supported in a study by Bellamri et al. from 2020 (41). Here macrophages were found to be one of the main producers of CXCL13 and to be dependent on IL-10 signaling (41). We observed two peaks *of Il10* mRNA expression in inflamed salivary gland tissue: one in the acute phase and one when B cells were observed in the early phases of TLS development. A similar pattern was observed in *Cxcl13* mRNA expression, indicating that, in addition to contributing to disease progression by promoting inflammation and tissue damage, salivary gland macrophages might also have a regulatory function and aid in the resolution of inflammation. Tissue-resident macrophages (41) and alternatively activated (M2) macrophages (43) together with regulatory B cells are known to produce IL-10 (44). IL-10 is an important component of the function of regulatory B cells and involved in controlling the inflammation process from an active to a resolution phase (44, 45).

In our study, the expression of CD206, a marker associated with M2-macrophages (19, 34, 46), was observed in cDC2, macrophages and patrolling monocytes in salivary gland tissue prior to infection. Interestingly, the relative frequency of CD206^+^ cells decreased during the early phases of acute inflammation before it increased again in the later stages of the inflammatory process. This biphasic pattern of CD206 expression suggests a nuanced role of these cells in the immune response. Initially, during the acute phase, the decrease in relative frequency of CD206 downregulation may reflect a shift towards a proinflammatory macrophage phenotype, which is necessary for the initial defense against pathogens and the establishment of TLSs. As inflammation progresses, the upregulation of *Cd206* mRNA in tissue and increase in CD206 expression among macrophages and cDC2 signifies a transition towards an anti-inflammatory or tissue-repairing state (19, 47). This is consistent with the observed peaks in IL-10 expression. The increase in CD206 expression during the resolution phase may thus represent a regulatory mechanism that facilitates the resolution of inflammation and maintenance of TLSs, potentially through the production of IL-10 and CXCL13. These findings underscore the dual role of macrophages in both the propagation and resolution of inflammation within the salivary glands of patients with primary Sjögren’s disease and highlight the importance of these cells in the complex interplay between immune activation and regulation in autoimmune disease pathology.

Flow cytometric analysis of the myeloid cell population revealed that the F4/80^+^ macrophages were positive for CX3CR1. The cDC2 population was also one of the main myeloid cell populations expressing CX3CR1, following a similar pattern of their expression of CD206 as F4/80^+^ macrophages. In studies of tissue injury in chronic inflammation, CX3CR1 expression among myeloid cells was linked to proinflammatory responses and associated with immune cell recruitment (15, 48, 49). In a study published in 2020, Koscso and colleagues examined the CX3CR1^+^ myeloid cell population in the aggregation of immune cells and induction of mucosal IgA in the gut (16). One of their main findings was the superior function of CX3CR1^+^ macrophages as antigen-presenting cells responsible for recruitment and activation of CD4^+^ T and B cells and later as contributing cells for initiating TLS development and controlling the local pathogen-specific IgA response (16). CX3CR1 expression in macrophages can be further linked to lymphoid neogenesis in salivary gland tissues, as CX3CR1 expression was observed in immune cell-infiltrated tissues from patients with primary Sjögren’s disease (50).

PDPN^+^ macrophages were located both in the parenchyma of the tissue and encapsulating the TLSs. From our flow cytometric analysis of the myeloid cell population, we observed that CX3CR1^+^PDPN^+^ expression increased in macrophages, cDC2 and monocytes in the initial stages of acute inflammation. However, only the macrophage population continued to express PDPN throughout the inflammatory process. PDPN expression in stromal cells have shown to be crucial for lymphoid tissue formation and expansion (5, 9, 14, 32, 51–53). The function of PDPN in macrophages is still not fully understood. In 2012, Kerrigan et al. showed that inflammatory macrophages express PDPN and activate platelets via CLEC-2 (54). However, several studies have linked PDPN and macrophages to stimulate lymphangiogenesis to facilitate the recruitment of lymphocytes in inflamed tissues caused by infection, cancer or chronic inflammation (17, 46, 54–59). Expansion of the lymphatic vascular network is one of the features involved in TLS development and function (60). We observed similar organization of PDPN^+^ macrophages in the two mouse models of Sjögren’s disease (20, 61). Thus, indicating that these macrophages might encapsulate the TLS structures and be involved in TLS development and function in both virus induced and chronic inflammation associated with autoimmune diseases.

Macrophages are important for the immune surveillance of the lymph nodes and spleen (62). Macrophages in these tissues can facilitate both immune initiation and control of immune responses, including the removal of apoptotic cells, clearance of pathogens, antigen presentation and cytokine production (62–65). Comparing the similarities of macrophages from the lymph nodes and spleen with macrophages observed in the salivary glands during TLS development might provide insights into the possible functions of macrophages in TLSs. The subcapsular sinus macrophages (SSM) in the lymph node encapsulate the organ and participate in lymph filtration and are one of the first immune cells that interact with lymph-borne pathogens and present antigens to B cells (66, 67). The medullary sinus macrophages (MSM) are efficient phagocytes and promoters of inflammatory response, with their abundant ability to present antigens (63, 66, 68–70). Phenotypically, the main differences between SSM and MSM is their expression pattern of F4/80 in mice, since both are known to be expressing CD169 (25, 70). SSM has a low F4/80 expression, while MSM express it strongly (66, 68, 70). From our studies, we can hypothesize that the macrophages we observed surrounding the TLSs might have similar functions as both SSM and MSM. However, further studies on the exact function of macrophages in TLSs are required.

In conclusion, our study sheds light on some of the dynamic roles of macrophages in the pathogenesis of Sjögren’s disease, particularly in the context of TLS development within salivary glands. Macrophage’s role in ectopic lymphoid neogenesis is multifaceted, potentially supporting the organization and survival of lymphoid cells within TLSs. The identification of distinct macrophage populations characterized by CX3CR1, PDPN, and CD206 expression potentially contributes to both the perpetuation of chronic inflammation and resolution of the disease. Their exact contribution to disease progression may depend on the balance between proinflammatory and anti-inflammatory activities as well as their interactions with other immune cells within the inflamed tissue. Our findings suggest that salivary gland macrophages in Sjögren’s disease might share functional similarities with their counterparts in lymph nodes. The analogy between macrophages surrounding the TLSs and subcapsular sinus macrophages (SSM) or medullary sinus macrophages (MSM) in lymph nodes invites further investigation into their shared and distinct roles in the immune system architecture and function. The intricate interplay between proinflammatory and regulatory macrophage functions within the salivary glands presents a nuanced picture of the autoimmune pathology in Sjögren’s disease. Focusing on the immune landscape of this disease might pave the way for targeting macrophages in therapeutic strategies with the potential to modulate disease progression.

## Data Availability

The original contributions presented in the study are included in the article/**Supplementary Material**. Further inquiries can be directed to the corresponding author.
