# Supplementary material for: Podoplanin expressing macrophages and their involvement in tertiary lymphoid structures in mouse models of Sjögren’s disease
Source: Front Immunol. 2024 Sep 17;15:1455238. doi: 10.3389/fimmu.2024.1455238 (PMC11442383; doi:10.3389/fimmu.2024.1455238)
Supplement: Supplementary file 1 [file DataSheet1.pdf]

## Supplementary materials and method

### Buffers

*Staining buffer for flow cytometry - PBS with 0,5%BSA and 2mM EDTA*

- 1L PBS pH 7.2
- 5g bovine serum albumin (BSA)
- 4mL 0.5M EDTA

*TBS wash buffer pH 7,5 (room temperature)*

- *25mM TRIS-HCL*
- *150mM NaCl*

*10 X Tris buffer (10X TBS):*

- 24 g Tris-HCl (Tris(hydroxymethyl)aminomethane)
- 88 g NaCl (Sodium Chloride)
- 800 ml MilliQ water
- Adjust pH 7.5 with HCl.
- Adjust water up to 1L.
- Dilute 1:10 to make 1X TBS.

*TBST wash buffer (room temperature)*

- 25mM TRIS-HCl, PH 7.5
- 150 mM NaCl
- 0.05% Tween 20 (v/v)
- Add 500µL Tween to 1L 1X TBS to make TBST.

*10X Citrate Buffer for Antigen Retrieval/Epitope retrieval*

- 29,4g Tri-sodium citrate (dihydrate)
- Adjust to pH 6 with HCl, adjust volume to 1L with MilliQ water.
- Dilute 1:10 to make 1X Citrate buffer.

**Supplementary Table 1: Antibodies and their dilution for antibody cocktail for flow cytometry**

| Panel | Marker | Fluorochrome conjugated | Clone | Product number/ Producent | Dilution in cocktail |
|---|---|---|---|---|---|
| **Panel 1 and 2** | CD3 | BV510 | 145-2C11 | 100353/BioLegend | 1:200 |
| **Panel 1 and 2** | CD19 | BV510 | 6D5 | 115546/ BioLegend | 1:200 |
| **Panel 1 and 2** | NK 1.1 | BV510 | PK136 | 108738/ BioLegend | 1:200 |
| **Panel 1 and 2** | F4/80 | APC | BM8 | 123116/ BioLegend | 1:100 |
| **Panel 1 and 2** | CD11c | BV650 | N418 | 117339/ BioLegend | 1:200 |
| **Panel 1 and 2** | CD11b | PE-Cy7 | M1/70 | 101216/ BioLegend | 1:200 |
| **Panel 1 and 2** | Ly6C | BV421 | HK1.4 | 128031/ BioLegend | 1:100 |
| **Panel 1 and 2** | MHC-II | PE-Dazzle 594 | M5/114.15.2 | 107648/ BioLegend | 1:300 |
| **Panel 1 and 2** | CX3CR1 | PerCP-Cy-5.5 | SA011F11 | 149010/ BioLegend | 1:200 |
| **Panel 1 and 2** | Ly6G | AF700 | 1A8 | 127622/ BioLegend | 1:50 |
| **Panel 1** | CD45 | APC-Fire750 | 30-F11 | 103154/ BioLegend | 1:400 |
| **Panel 1** | PDPN | FITC (AF488) | 8.1.1 | 53-5381-82/Thermo scientific | 1:200 |
| **Panel 1** | CCR2 | BV605 | SA203G11 | 150615/BioLegend | 1:400 |
| **Panel 1** | Siglec F | PE | S17007L | 155506/ BioLegend | 1:200 |
| **Panel 1** | CD115 | BV711 | AFS98 | 135515/ BioLegend | 1:100 |
| **Panel 2** | CD45 | BV711 | 30-F11 | 103147/ BioLegend | 1:200 |
| **Panel 2** | CD80 | BV605 | 16-10A1 | 104729/ BioLegend | 1:400 |
| **Panel 2** | CD86 | AF488 | GL-1 | 105018/ BioLegend | 1:200 |
| **Panel 2** | CD206 | PE | C068C2 | 141706/ BioLegend | 1:200 |
| **Panel 2** | CD40 | APC-Fire 750 | 3/23 | 124632/ BioLegend | 1:100 |

**Supplementary Table 2: Antibodies used for immunofluorescence labelling of salivary gland cryo-sections.**

| | Primary antibody cocktail | | | Corresponding secondary antibody cocktail | | |
|---|---|---|---|---|---|---|
| | **Antibody (clone)** | **Product number/ Producent** | **Concentration of antibodies and incubation time** | **Antibody (AF* fluorochrome conjugate)** | **Product number/ Producent** | **Concentration of antibodies and incubation time** |
| **F4/80, PDPN and CD206 staining** | F4/80 | 122603/ BioLegend | 1:200, 1hour | Goat anti-rat IgG (AF546) | A11081/ Invitrogen, Thermo Fisher Scientific | 1:1000, 30min |
| | PDPN | 14-5381-85/ Invitrogen, Thermo Fisher Scientific | 1:75, 1hour | Goat anti-Syrian hamster IgG (AF488) | A21110/ Invitrogen, Thermo Fisher Scientific | 1:1000, 30min |
| | CD206 | AB64693/ Abcam | 1:750, 1hour | Goat anti-Rabbit IgG (AF647) | A21245/ Invitrogen, Thermo Fisher Scientific | 1:1000, 30min |
| **CD45, B220 and CD3 staining** | B220 | MAB 1217/ RD System | 1:800, 30min | Goat anti-rat IgG (AF546) | A11081/ Invitrogen, Thermo Fisher Scientific | 1:1000, 30min |
| | CD3 | A0452/ DAKO | 1:400, 30min | Goat anti-Rabbit IgG (AF488) | A11034/ Invitrogen, Thermo Fisher Scientific | 1:2000, 30min |
| | CD45-APC** | 17-0451-82/ Invitrogen, Thermo Fisher Scientific | 1:50, 1hour | NA | NA | NA |

*AF; Alexa Fluor™

**CD45-APC; Fluorochrome direct conjugated to antibody, incubation of this antibody was performed in a separate step after B220/CD3 antibody incubation and their corresponding secondary antibody incubation.

**Supplementary Table 3: Overview of antibodies, their concentration and OPAL fluorophore applied.**

| Staining order | Antibody | Product number/ Producent | Concentration of antibodies and incubation time | Polymer HRP detection kit | OPAL Fluorophore |
|---|---|---|---|---|---|
| 1 | PDPN | 14-5381-85/ Invitrogen, Thermo Fisher Scientific | 1:250, 30 min | Polink-2 Plus HRP Anti- Syrian Hamster DAB Detection Kit (D86-18, Golden Bridge International) | OPAL 570 (1:100) SKU FP1488001KT / Akoya Biosciences |
| 2 | F4/80 | AB111101/ Abcam | 1:50, Over night | Rabbit pAb Envision+ system- HRP/DAB anti rabbit detection kit (K4003, DAKO) | OPAL 520 (1:100) SKU FP1487001KT / Akoya Biosciences |
| 3 | CD206 | AB64693/ Abcam | 1:1000, 30min | Rabbit pAb Envision+ system- HRP/DAB anti rabbit detection kit (K4003, DAKO | OPAL 690 (1:100) SKU FP1497001KT / Akoya Biosciences |
| 4 | CX3CR1 | AB217291/ Abcam | 1:250, 30min | Rabbit pAb Envision+ system- HRP/DAB anti rabbit detection kit (K4003, DAKO | OPAL 780 (1:50) SKU FP1501001KT / Akoya Biosciences |
| 5 | Opal DAPI | SKU FP1490/ Akoya Biosciences | 9µL DAPI + 100µL 1X TBS, 30min | | |

**Supplementary Table 4: Overview over NZBW-F1 mice.**

| | | dsDNA autoantibody | Age | Proteinuria |
|---|---|---|---|---|
| n(AB-)=3 | J01 | AB- | 22wo | Neg |
| | J02 | AB- | 25 wo | Neg |
| | J04 | AB- | 25 wo | Neg |
| n(AB+)=4 | J03 | AB+ | 25 wo | Neg |
| | J11 | AB+ | 33 wo | Neg |
| | J18 | AB+ | 34 wo | Pos |
| | J13 | AB+ | 27 wo | Pos |

**Supplementary Table 5: TaqMan probes for qPCR of mRNA from Thermo Fisher Scientific**

| Gene | Cat. Nr | Gene | Cat. Nr | Gene | Cat. Nr |
|---|---|---|---|---|---|
| *Ccl21* | Mm03646971_gh | *Ifny* | Mm99999071-m1 | *Mx1* | Mm00487796-m1 |
| *Cd169* | Mm00488332_m1 | *Il10* | Mm99999062_m1 | *Pdpn* | Mm01348912_g1 |
| *Cd206* | Mm00711660_m1 | *Il18* | Mm00434226-m1 | *Tbp* | Mm00446973_m1 |
| *Cx3cr1* | Mm02620111_s1 | *Il1B* | Mm00434228-m1 | *TgfB* | Mm01178820_m1 |
| *Ccxcl13* | Mm00444534_m1 | *LtB* | Mm00434774_g1 | *Tnf* | Mm00443258-m1 |
| *Foxp3* | Mm00475162-m1 | *LtBr* | Mm00440235_m1 | | |

## Supplementary figures

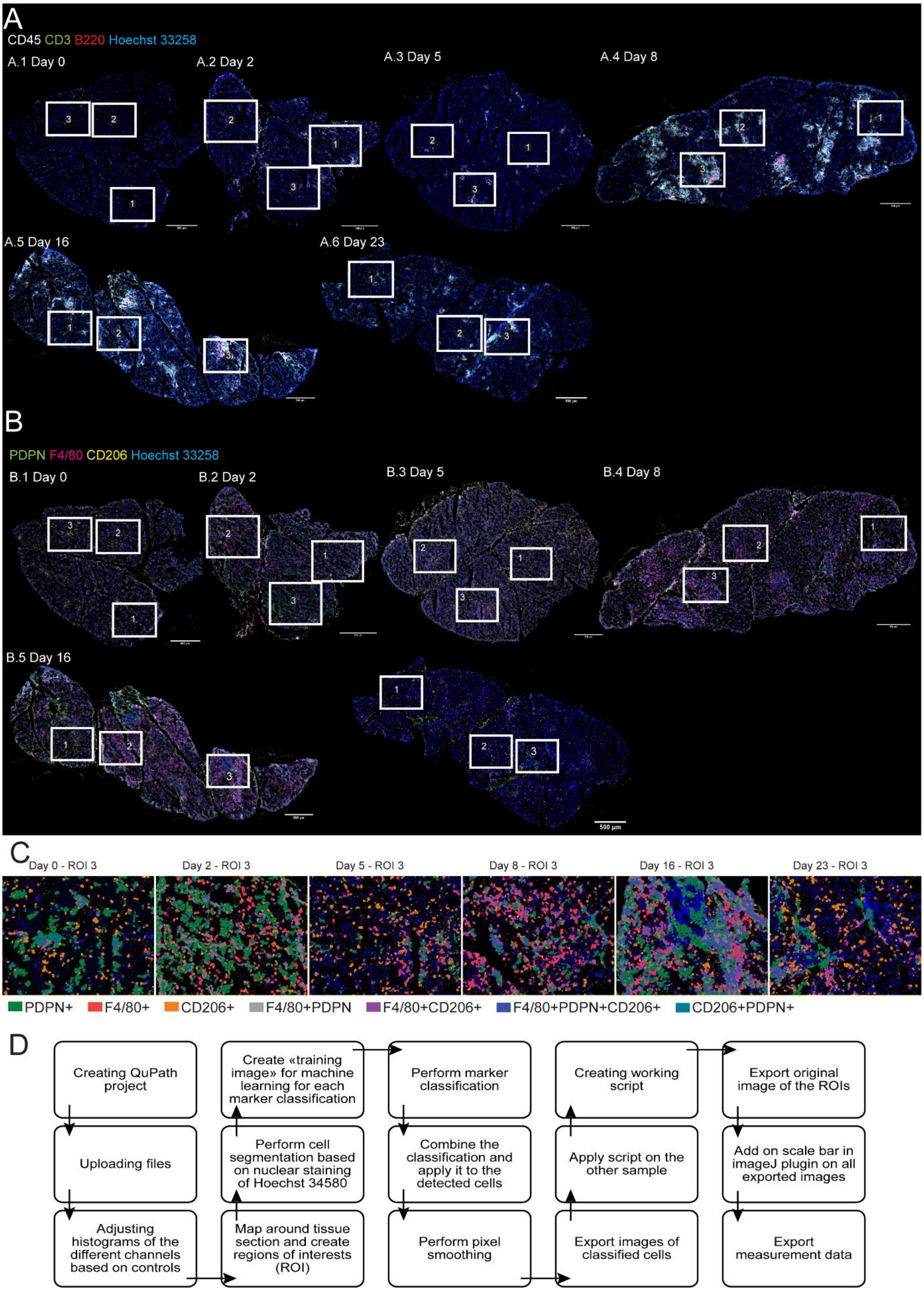

**Supplementary figure 1. Immunofluorescence labelling and segmentation analysis.**

Representative overview images over the A] CD45, CD3 and B220 labelling and B] PDPN, F4/80 and CD206 labelling of the salivary gland serial cryosections, including the localization of the three regions of interest (ROI). The different ROI are marked with numbered white boxes, where each number correspond to one of the three ROIs. 1 indicates ROI1 – Region with low inflammation, 2 indicates ROI2 – Region with inflammation, and 3 indicate ROI3 – Region with T and B cell aggregation. The aggregation of B and T cells, together with the infiltration of non-lymphocyte CD45+ cells, was used to allocate different ROIs. Scale bare C] Segmentation analysis of F4/80, PDPN and CD206 IF labelling. Segmentation is shown as an overlay of the original image. D] Analysis pipeline of fluorescence labelled salivary gland sections with QuPath V-0.5.1.

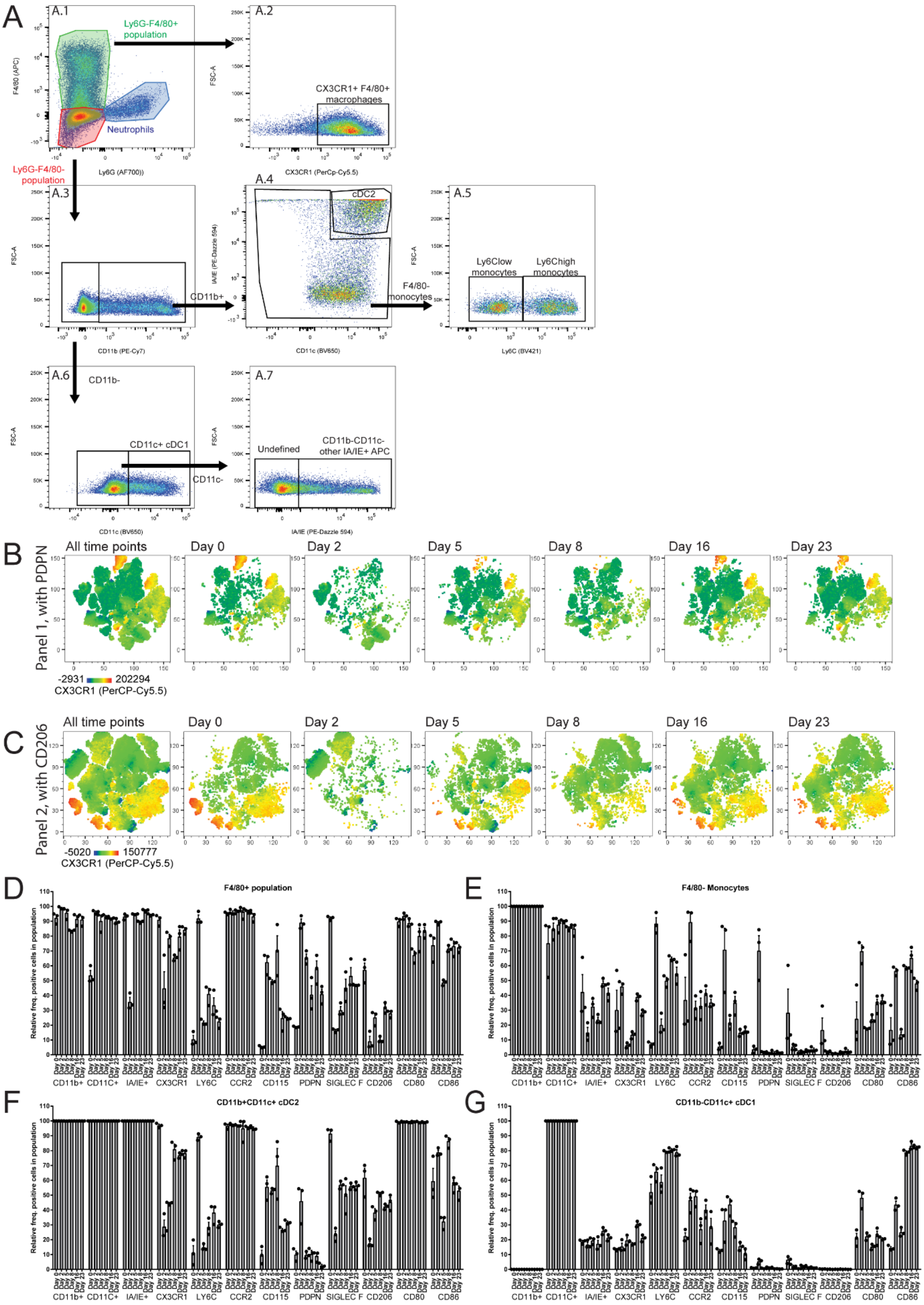
A
A.1
Ly6G-F4/80+ population
Neutrophils
Ly6G-F4/80- population
F4/80 (APC)
Ly6G (AF700)
A.2
CX3CR1+ F4/80+ macrophages
FSC-A
CX3CR1 (PerCp-Cy5.5)
A.3
CD11b+
CD11b (PE-Cy7)
A.4
cDC2
F4/80- monocytes
IA/IE (PE-Dazzle 594)
CD11c (BV650)
A.5
Ly6Clow monocytes
Ly6Chigh monocytes
Ly6C (BV421)
A.6
CD11b-
CD11c+ cDC1
CD11c-
CD11c (BV650)
A.7
Undefined
CD11b-CD11c- other IA/IE+ APC
IA/IE (PE-Dazzle 594)
B
Panel 1, with PDPN
All time points
Day 0
Day 2
Day 5
Day 8
Day 16
Day 23
-2931
202294
CX3CR1 (PerCP-Cy5.5)
C
Panel 2, with CD206
All time points
Day 0
Day 2
Day 5
Day 8
Day 16
Day 23
-5020
150777
CX3CR1 (PerCP-Cy5.5)
D
F4/80+ population
E
F4/80- Monocytes
F
CD11b+CD11c+ cDC2
G
CD11b-CD11c+ cDC1
Relative freq. positive cells in population
CD11b+
CD11C+
IA/IE+
CX3CR1
LY6C
CCR2
CD115
PDPN
SIGLEC F
CD206
CD80
CD86

**Supplementary figure 2. Flow gating strategy over the myeloid cell population and tSNE plot analysis.**

A] Gating strategy for identifying myeloid cell populations in the salivary gland during LucAdV5 infection. Shown is a representative gating strategy for myeloid cell populations from the salivary gland based on combining all time points. The myeloid cell population was defined as live $CD45^+$, $CD19^-$, $CD3^-$ and $NK1.1^-$ cells and were gated according to this approach: FSC-A VS. SSC-A, FSC-H VS. FSC-A, FSC-A VS CD45, FSC-A VS live/dead and dump channel (CD19, CD3, NK.1.1). The following populations are located in the respective gates; A.1] Neutrophils and $F4/80^+$ macrophage/monocyte population, A.2] $CX3CR1^+$ $F4/80^+$ macrophages/monocytes, A.4] $CD11b^+CD11c^+$ $IA/IE^+$DC2 and Monocytes, A.5] Ly6Chigh and Ly6Clow monocytes, A.6] $CD11b^-CD11c^+$ DC1, A.7] Other $CD11b^-IA/IE^+$ antigen-presenting cells (APC) and $CD11b^-F4/80^-CD11c^-$ IA/IE-undefined cell population. tSNE-plot with heatmap of CX3CR1 expression in B] panel 1 – containing PDPN and in C] panel 2 -containing CD206. The relative frequency of the following markers: CD11b, CD11c, IA/IE (MHC-II), CX3CR1, Ly6C, CCR2, CD115, PDPN, Siglec-F, CD206, CD80 and CD86 were analyzed by flow cytometry in the different myeloid cell populations D] $F4/80^+$ macrophages, E] $F4/80^-$ monocytes, F] $CD11b^+CD11c^+$ cDC2 and G] $CD11b^-CD11c^+$ cDC1.

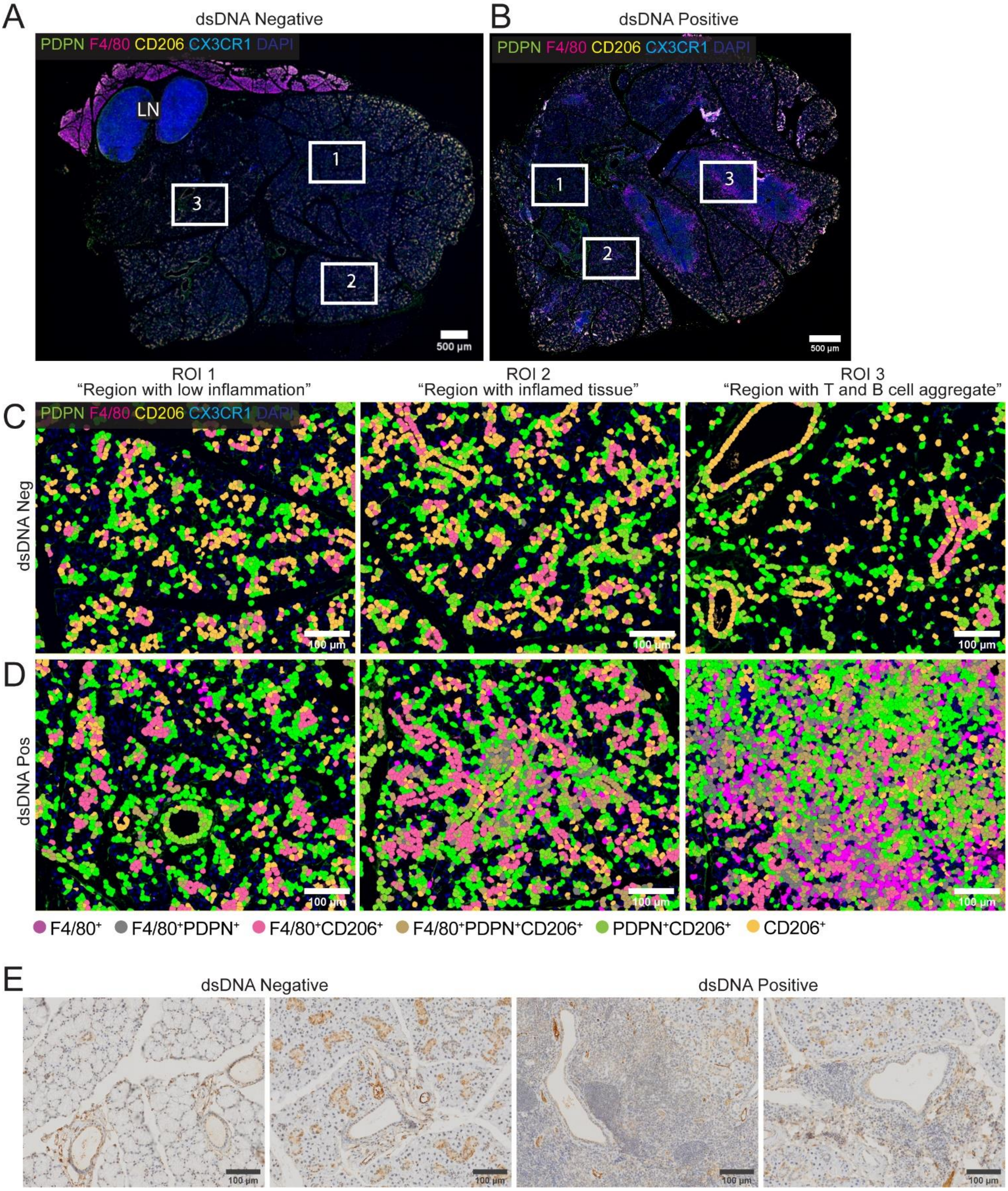
A
dsDNA Negative
PDPN F4/80 CD206 CX3CR1 DAPI
LN
1
2
3
500 µm
B
dsDNA Positive
PDPN F4/80 CD206 CX3CR1 DAPI
1
2
3
500 µm
ROI 1
"Region with low inflammation"
ROI 2
"Region with inflamed tissue"
ROI 3
"Region with T and B cell aggregate"
C
PDPN F4/80 CD206 CX3CR1 DAPI
dsDNA Neg
100 µm
D
dsDNA Pos
100 µm
F4/80+ F4/80+PDPN+ F4/80+CD206+ F4/80+PDPN+CD206+ PDPN+CD206+ CD206+
E
dsDNA Negative
dsDNA Positive
100 µm

**Supplementary figure 3. Immunofluorescence labelling and segmentation analysis of salivary glands from NZBW-F1.**

Representative overview images over the PDPN, F4/80, CD206 and CX3CR1 labelling of the NZBW-F1 salivary gland sections from anti-dsDNA autoantibody A] negative and B] positive mice. These overview images include the localization of the three regions of interest (ROI). Segmentation analysis of F4/80, PDPN and CD206 IF labelling. Segmentation is shown as an overlay of the original image of C] anti-dsDNA autoantibody negative and D] anti-dsDNA autoantibody positive mice. Labelling of CX3CR1 was not added to the segmentation analysis. E] Representative immunohistochemistry images of CX3CR1 labelling of salivary gland tissues.
